# Accessing inoculation methods of maize and wheat with *Azospirillum brasilense*

**DOI:** 10.1186/s13568-015-0171-y

**Published:** 2016-01-13

**Authors:** Josiane Fukami, Marco Antonio Nogueira, Ricardo Silva Araujo, Mariangela Hungria

**Affiliations:** Embrapa Soja, Cx. Postal 231, Londrina, Paraná 86001-970 Brazil; Department of Biochemistry and Biotechnology, Universidade Estadual de Londrina (UEL), C.P. 10.011, Londrina, Paraná 86051-990 Brazil; Total Biotecnologia Indústria e Comércio Ltda, Rua Emílio Romani 1190, CIC, Curitiba, Paraná 81460-020 Brazil

**Keywords:** *Azospirillum brasilense*, Biological nitrogen fixation, Leaf spray, Plant growth promoting bacteria, Soil spray, *Triticum aestivum* L., *Zea mays* L.

## Abstract

The utilization of inoculants containing *Azospirillum* is becoming more popular due to increasing reports of expressive gains in grain yields. However, incompatibility with pesticides used in seed treatments represents a main limitation for a successful inoculation. Therefore, in this study we searched for alternatives methods for seed inoculation of maize and wheat, aiming to avoid the direct contact of bacteria with pesticides. Different doses of inoculants containing *Azospirillum brasilense* were employed to perform inoculation in-furrow, via soil spray at sowing and via leaf spray after seedlings had emerged, in comparison to seed inoculation. Experiments were conducted first under greenhouse controlled conditions and then confirmed in the field at different locations in Brazil. In the greenhouse, most parameters measured responded positively to the largest inoculant dose used in foliar sprays, but benefits could also be observed from both in-furrow and soil spray inoculation. However, our results present evidence that field inoculation with plant-growth promoting bacteria must consider inoculant doses, and point to the need of fine adjustments to avoid crossing the threshold of growth stimulation and inhibition. All inoculation techniques increased the abundance of diazotrophic bacteria in plant tissues, and foliar spray improved colonization of leaves, while soil inoculations favored root and rhizosphere colonization. In field experiments, inoculation with *A. brasilense* allowed for a 25 % reduction in the need for N fertilizers. Our results have identified alternative methods of inoculation that were as effective as the standard seed inoculation that may represent an important strategy to avoid the incompatibility between inoculant bacteria and pesticides employed for seed treatment.

## Introduction

The bacterial genus *Azospirillum* encompasses bacteria associated with various plant species such as maize (*Zea mays* L.), wheat (*Triticum aestivum* L.) and sugarcane (*Saccharum* spp.) (Swedrzyńska and Sawicka 2001; Hungria et al. 2010; Moutia et al. 2010; Piccinin et al. 2011). *Azospirillum* spp. are by far the best-studied plant growth-promoting bacteria (PGPB) (Bashan and de-Bashan 2010). They are believed to stimulate plant growth by an array of mechanisms including, but not restricted to, production and secretion of phytohormones (Tien et al. 1979; Bottini et al. 1989), increase of nutrient availability (Rodriguez et al. 2004; Bashan and de-Bashan 2005; Hungria et al. 2010), and biological nitrogen fixation (BNF) (de-Bashan et al. 2012). In addition, *Azospirillum* spp. have been implicated in increasing plant resistance to pathogens (Romero et al. 2003; Tortora et al. 2011). Due to the wide array of mechanisms proposed for stimulation of plant growth by *Azospirillum* spp., Bashan and de-Bashan (2010) proposed a theory of multiple mechanisms that might act either in a cumulative or sequential pattern.

The analysis of results from a large number of field trials with various non-legume crops, conducted worldwide over 20 years, under different soil and weather conditions, has demonstrated that yield increases of up to 30 % could be obtained 70 % of the time (Okon and Labandera-Gonzalez 1994) in response to inoculation with *Azospirillum*. In addition, an extensive evaluation of wheat inoculated with a commercial liquid *Azospirillum brasilense* formulation at 297 locations in Argentina has demonstrated positive responses of up to 6 % increases in yield in 70 % of the cases, depending on the experimental conditions (Diaz-Zorita and Fernández-Canigia 2009). In Brazil, Hungria et al. (2010) observed increases of up to 30 and 18 % in the grain yields of maize and wheat, respectively, inoculated with elite strains of *A. brasilense* in field trials. In addition, there are also reports of grain yield increases by co-inoculation of legumes with *A. brasilense* and rhizobia, e.g. in soybean (*Glycine max* (L.) Merr.) and common bean (*Phaseolus vulgaris* L.) (Hungria et al. 2013, 2015).

Even though field inoculation with *Azospirillum* may promote crop yield increases, limitations related to strain compatibility with chemicals employed for seed treatment—mostly pesticides—may be expected (Puente et al. 2008), as is the case with other PGPB such as *Bradyrhizobium* (Campo et al. 2009). There is little information available about the toxicity of pesticides employed for seed treatment towards non-target microorganisms and PGPB, and their various modes of action may affect different aspects of such beneficial microorganisms, making it difficult to infer about compatibility between products and inoculants (Yang et al. 2011). Some chemicals have been shown to be very harmful to rhizobia (Hungria et al. 2005; Campo et al. 2009) and either harmless (Elslahi et al. 2014) or very toxic (Mohiuddin and Mohammed 2013) to *Azospirillum.*

Partial or total replacement of chemical fertilizers with PGPB may not only reduce costs, but also help to mitigate the negative environmental impacts of agricultural activities. There is much knowledge about formulations and inoculation technologies with PGPB (Bashan et al. 2014), but further studies are necessary to evaluate the ease and viability of large-scale inoculation strategies, taking into account that sowing is a critical phase of the agricultural activity due to weather and season constraints for each crop. In addition, since seed treatment with chemicals will continue to be practiced, and until more information on this subject is available, inoculation strategies should try to avoid damage to the bacteria. The hypothesis of our study is that it is possible to find alternatives methods to seed inoculation of cereals with *A. brasilense*, reducing or avoiding the negative impacts of chemicals applied to the seeds.

## Materials and methods

### Inoculants and inoculation methods

Inoculants consisted of a mixture of strains CNPSo 2083 (=Ab-V5) and CNPSo 2084 (=Ab-V6) of *A. brasilense* (from the Collection of Diazotrophic and Plant Growth-Promoting Bacteria of Embrapa Soja, WFCC # 1213, WDCM # 1054). Both strains derived from a selection program that evaluated N_2_-fixing capacity in vitro and under field conditions (Hungria et al. 2010). The strains were shown to be highly efficiency in promoting growth of wheat and maize in several trials in Brazil, mainly due to their capacity of producing plant hormones and increasing root growth and nutrients uptake. Both strains are currently employed for commercial production of *Azospirillum* inoculants in Brazil.

Four methods of inoculation were compared: (1) standard seed inoculation (SI) – control treatment; (2) inoculation in the planting furrow at sowing (IPF); (3) leaf spray inoculation at the V2.5 stage of the maize plant growth cycle (Hickman and Shroyer 1994) or 3rd tiller (Large 1954) for wheat (ILS); and (4) spray inoculation on the soil surface at the V2.5 stage of the maize plant growth cycle (Hickman and Shroyer 1994) or 3rd tiller (Large 1954) for wheat (ISS).

For the maize crop 1 dose of inoculant corresponded to the application of 1.0 × 10^5^ cells seed^−1^ for seed inoculation (SI) and in-furrow (IPF) and to 1.0 × 10^5^ cells plant^−1^ for leaf spray (ILS) and soil spray (ISS). For the wheat crop 1 dose of inoculant corresponded to the application of 1.74 × 10^4^ cells seed^−1^ for SI and IPF and to 1.74 × 10^4^ cells plant^−1^ for ILS and ISS. Different doses of inoculant were evaluated.

### Greenhouse experiments

First, greenhouse experiments were performed aiming at screening the treatments for the field experiments and to obtain preliminary results about diazotrophic populations in plant tissues with different types of inoculation. We did not repeat the greenhouse experiments because, once the preliminary results were obtained, we went straight to the field experiments.

For the greenhouse experiments, both maize and wheat were grown in 3 kg-pots filled with soil collected from the 0–20 cm top layer of an Oxisol (Latossolo Bruno by the Brazilian classification) (Embrapa 2006) from Ponta Grossa (25°13′ S; 50°1′ W), Paraná State, Brazil. Soil characteristics are presented in Table 1. Soil acidity and base saturation were corrected by adding 6 g of lime pot^−1^ (equivalent to 4 t ha^−1^). Fertilization was accomplished by the addition of 100 mL micronutrient solution (H_3_BO_3_, 7.5 mg; Na_2_MoO_4_·2H_2_O, 0.3 g; CoSO_4_·7H_2_O, 0.0045 mg) per pot and 200 mL of a phosphorus solution to provide 550 mg K_2_HPO_4_ per pot. Soils were poor on P, therefore we had to supply P, and micronutrients were added as recommended for the crops in this type of soil.

**Table 1:** Chemical (before liming) and granulometric characteristics of the soil employed for greenhouse experiments

Chemical										Granulometric		
P (mg dm^−3^)	pH (CaCl_2_)	Al (cmol_c_ dm^−3^)	H + Al (cmol_c_ dm^−3^)	Ca + Mg (cmol_c_ dm^−3^)	K (cmol_c_ dm^−3^)	SB (cmol_c_ dm^−3^)	CEC (cmol_c_ dm^−3^)	BS (%)	C g dm^−3^	Clay (%)	Silt (%)	Sand (%)
3.70	4.82	0.12	5.68	3.44	0.25	3.69	9.37	39.4	8.2	24.70	6.82	68.48

*SB* sum of bases (Ca + Mg + K), *CEC* cation exchange capacity (SB + H + Al), *BS* bases saturation = [(K + Ca + Mg)/CEC] × 100

Pots were arranged in a completely randomized design with 18 treatments and five replicates. Treatments consisted of combinations of varying doses of N fertilizer (100 % and 75 % N) and inoculants (1× and 2.5×), and different methods of inoculation (SI, IPF, ILS, and ISS).

When maize was planted, each pot received three seeds of the BRS 3010 hybrid (Embrapa). Seeds, which were not surface disinfected in order to mimic field conditions, were treated with fungicide Maxim™XL [active ingredient (a.i.: 2.5 % fludioxonil); 1.0 % metalaxyl − M] and insecticides Actellic™500 CE (a.i.: 50 % methyl pirimifos) and K-Obiol™25 CE (a.i.: 2.5 % deltametrine), according to technical recommendations for the crop in Brazil. Five days after emergence (DAE), one seedling was removed, leaving two plants per pot.

Wheat seeds were treated with Maxim™X, and each pot received four non-disinfected seeds of cultivar BRS Gaivota (Embrapa), leaving only two seedlings per pot at 6 DAE.

Seed inoculation was performed 1 h before sowing by evenly coating seeds with the appropriate amounts of inoculants. For the in-furrow inoculation, inoculant diluted in sterile distilled water [1:500 (v:v), for maize; 1:3000 (v:v) for wheat] was applied to the soil with the help of an adjustable pipetor, immediately before sowing, to simulate the action of a planting device. Here it is worth mentioning that although the dilution with water might not be ideal in terms of osmotic effect for the bacterium, we wanted to follow what the farmer does under field conditons, where he mixes the inoculant with water. For leaf and soil surface spray inoculation, an aerograph atomizer was employed to mimic the action of a spraying equipment. For leaf spray inoculation, the soil surface was covered with aluminum foil in order to avoid that the inoculant reached the soil. For soil surface spray inoculation, plant shoots were covered with plastic bags to make sure that the inoculant reached only the soil surface.

For the final volume of liquid for both leaf and soil surface spray inoculation was 1 mL (water + inoculant) per pot containing two plants, and inoculants were diluted with sterile distilled water at 1:1000 (v:v) and 1:7500 (v:v) for spraying maize and wheat, respectively. Foliar and soil spray inoculations of pots containing maize plants were performed 11 DAE, during the V2.5 vegetative stage (Hickman and Shroyer 1994). In the case of wheat, inoculation took place 17 DAE, when plants were at tiller stage 3, according to the scale of Feeks and Large (Large 1954).

- For the maize plants, N was supplied as NH_4_NO_3_ in order to provide 120 (100 %) and 90 (75 %) kg N ha^−1^. N was applied in equal amounts every 8 days.
- For the wheat plants, N was supplied as NH_4_NO_3_ in order to provide 80 (100 %) and 60 (75 %) kg N ha^−1^. N was applied in equal amounts every 8 days.
- Average temperatures in the greenhouse during the experiments were of 29/16 °C (day/night) for maize, and 27/15 °C (day/night) for wheat; light inside the greenhouse was very close to the regular light, with a decrease of only 10 % of the radiation.

Plants from maize treatments were harvested 52 DAE (end of vegetative stage) for measurements of plant components. Plants from wheat treatments were harvested 54 DAE (end of vegetative stage) for measurements of plant components.

Before plants were harvested, chlorophyll content (CC) was determined according to Kaschuk et al. (2009) and based on the SPAD (Soil Plant Analysis Development) index, with readings taken from the lowermost third of the +3 (Trani et al. 1983) leaf for maize, and of the last fully expanded leaf for wheat.

Biometric parameters such as plant height (cm; PH) and culm diameter (mm; CD) of maize plants were determined with the aid of a digital caliper. In the case of wheat, the number of tillers (NT) was determined. For both crops, root volume was determined by measuring water displacement caused by immersion of the root systems in a graduate cylinder with a known volume of water.

Shoot dry weight (SDW) and root dry weight (RDW) were determined after drying plant material at 60 °C for approximately 72 h, until constant weight. Dry shoots were then ground (18 mesh) and subjected to sulfuric digestion to determine total shoot N by the salicylate green method (Searle 1984).

The populations of diazotrophic bacteria inside the leaves, roots, and rhizosphere were estimated by the most probable number (MPN) technique, as described before (Hungria and Araujo 1994; Döbereiner et al. 1995), from dilutions (10^5^ to 10^9^) of soil or homogenized tissues. Diazotrophic populations in leaves and roots were always evaluated in superficially disinfected tissues (Döbereiner et al. 1995). We used the classical MPN method in our study because we wanted to be sure that the same strains would be evaluated in the field experiments. Brazilian soils carry very high populations of *Azospirillum* (usually >10^4^ cells g^−1^), and there are so far no specific probes capable of distinguishing CNPSo 2083 and CNPSo 2084 from indigenous strains. The surface disinfection of leaves and roots should allow to access bacteria inside the tissues, including both obligatory and facultative endophytes, while the counting of bacteria in the rhizosphere would estimate the population of associative bacteria (Döbereiner et al. 1995). It is worth mentioning that despite the limitations of the NMP method, our goal was to have an indication if the bacteria could, or could not colonize tissues and to establish in the rhizosphere.

### Field experiments

#### Sites descriptions

Three field trials with maize and one with wheat were performed in the 2012/2013 (maize, summer crop) and 2013 (wheat, winter crop) cropping seasons. The experimental sites were located in Cachoeira Dourada, Luis Eduardo Magalhães, and Ponta Grossa (maize) and Ponta Grossa (wheat) (Table 2). Before sowing of the summer crop (maize), 20 subsamples were collected from the top (0–20 cm) layer of soil from each location, were dried (40 °C, 48 h) and sieved (2 mm) to prepare a composite sample, which was analyzed to determine chemical and granulometric properties (Table 3) (Klute 1986; Sparks 1996), as previously described (Hungria et al. 2006).

**Table 2:** Agronomic and climatic information about the sites where the field experiments were planted

Site	Altitude (m)	Soil type^a^	Climate^b^	Plant genotypes^c^	Sowing	Spacing (m)	Number of seeds m^−1^	Plant population ha^−1^	Date of harvest	Area harvested (m^2^)
Cachoeira Dourada (18º29′31″ S; 49º28′29″ W)	459	Latossolo Vermelho Distrófico	*Cwa*	Maize hybrid 2B707 HX	14/11/2012	0.8	6	75,000	15/05/2013	7.2
Luis E. Magalhães (12º05′31″ S; 45º48′18″ W)	720	Latossolo Amarelo Distrófico	*Aw*	Maize hybrid 2B707 HX	24/11/2012	0.8	5	75,000	20/04/2013	7.2
Ponta Grossa (25º13′ S; 50º1′ W)	880	Latossolo Vermelho-Escuro Distrófico	*Cbf*	Maize hybrid P4285 H	05/12/2012	0.5	4	82,000	17/04/2013	8.0
				Wheat cultivar BRS Pardela	25/06/2014	0.17	65	325,000	31/10/2013	7.2

^a^Soil classification according to Embrapa (2006); all oxisols

^b^Kööppen’s climatic classification

^c^Maize hybrid 2B707 HX (Down AgroScience), Maize hybrid P4285 H (Pioneer) and wheat cultivar BRS Pardela (Embrapa)

**Table 3:** Chemical and granulometric characteristics of 0–20 cm layer of the soils at the locations where field experiments were planted

Site	Chemical										Granulometric		
	P (mg dm^−3^)	pH (CaCl_2_)	Al (cmol_c_ dm^−3^)	H + Al (cmol_c_ dm^−3^)	Ca + Mg (cmol_c_ dm^−3^)	K (cmol_c_ dm^−3^)	SB (cmol_c_ dm^−3^)	CEC (cmol_c_ dm^−3^)	BS (%)	C (g dm^−3^)	Clay (%)	Silt (%)	Sand (%)
Cachoeira Dourada	4.50	5.25	0	4.05	4.97	0.35	5.32	9.37	56.8	28.40	58.65	18.55	22.80
Luis E. Magalhães	21.34	5.53	0	0.83	1.20	0.04	1.24	2.07	59.9	6.35	10.95	1.05	88.00
Ponta Grossa	2.80	4.90	0.17	4.75	4.85	0.23	5.08	9.83	51.7	30.50	23.80	3.00	73.20

All analyses were performed before sowing

*SB* sum of bases (Ca + Mg + K), *CEC* cation exchange capacity (SB + H + Al), *BS* bases saturation = [(K + Ca + Mg)/CEC] × 100

#### Experimental design and procedures

All field trials were set in a completely randomized block design comprising 11 treatments, with six replicates. Experiments were planted with commercial seeds and the maize and wheat genotypes used are listed in Table 2. Seeds were treated with fungicide Maxim^®^XL and insecticides Actellic^®^500 CE (a.i.: 50 % methyl pirimifos) and K-Obiol^®^25 CE (a.i.: 2.5 % deltametrine).

Maize plots measured 4 m (wide) × 8 m (long). The experiment received 300 kg ha^−1^ of NPK (08-20-20) fertilizer in the sowing furrow, to provide 24 kg N ha^−1^ immediately before sowing. Thirty-five DAE, plants received complementary doses of urea-N fertilizer, corresponding to 75 % N (67.5 kg ha^−1^) and 100 % N (90 kg ha^−1^) of the amount prescribed for the maize crop in Brazil. Therefore, when we mention 75 % of the dose, that refers to 75 % of the complementary dose of N-fertilizer, as the basal level of N was applied to all treatments. In Cachoeira Dourada, *Spodoptera frugiperda* insects were controlled with lufenuron (15 g a.i. ha^−1^).

Wheat plots measured 4.6 m (wide) × 6 m (long). The experiment received 70 kg P ha^−1^ (supplied as super triple phosphate), 40 kg K ha^−1^ (supplied as potassium chloride) and either 24 kg N (urea) ha^−1^ at sowing plus 67.5 kg N ha^−1−^as side dress (75 % N treatment) or 24 kg N ha^−1^ at sowing plus 90 kg N ha^−1^ as side dress (100 % N treatment). Therefore, again, when we mention 75 % of the dose, it refers to 75 % of the complementary dose of N-fertilizer, as the basal level of N was applied to all treatments.

Inoculation in the field compared standard seed inoculation (SI; control treatment) with the application of one, two or four doses of inoculants in the planting furrow (IPF), and leaf (ILS) and soil (ISS) spray inoculations, in the same concentrations specified in the greenhouse experiments

In the case of inoculation in-furrow, as well as of foliar and soil spray inoculations, inoculants were diluted with water to a final volume of 150 L ha^−1^. Seed and in-furrow inoculations were performed at sowing, whereas leaf and soil spray inoculation took place when maize plants were at the V2.5 (Hickman and Shroyer 1994) vegetative stage and when wheat plants were at tiller stage 3 (Large 1954). Spray applications were performed with a costal spray equipament (Herbicat), with air induction plan spray (VI-110.015), pression of 45 pounds, adjusted to the application of medium drops (200–400 µm). Other pertinent agronomic information about the experiments are presented in Table 2.

For maize, shoot dry weight (SDW), leaf N content (NC), total N in shoots (TNS), and grain yield at 13 % humidity (Y) were determined. For SDW, five plants were collected per plot 56, 61, and 30 DAE, in Cachoeira Dourada, Ponta Grossa and Luis Eduardo Magalhães, respectively. In addition, 15 leaves (middle third section of each leaf without the main nerve) were taken from each plot for determination of NC and TNS at 92, 96, and 84 DAE in Cachoeira Dourada, Luis Eduardo Magalhães, and Ponta Grossa, respectively. In the case of wheat, only grain yield at 13 % humidity was determined.

### Statistical analyses

Data obtained from each experiment were first evaluated for normality and variance homogeneity, followed by the analysis of variance (ANOVA). In the case of greenhouse experiments, when *p* ≤ 0.05 was confirmed by the F test, Duncan’s post hoc multiple range test at *p* ≤ 0.05 was employed for multiple comparisons, followed by Dunnett’s test (*p* ≤ 0.05) for the comparisons of means relative to the control treatment. For the field experiment Duncan’s post hoc multiple range test at *p* ≤ 0.05 was employed for multiple comparisons (SAS Institute 2001).

## Results

### Greenhouse experiment with maize

Shoot dry weight (SDW) was significantly increased (27 %) by soil spray inoculation (ISS) at the V2.5 stage with 2.5 doses of inoculant in addition to complementary fertilization with 75 % of the recommended side-dress N, when compared to 75 % N fertilization alone (Table 4). When the same amount of inoculant was at planting in-furrow (IPF), and 75 % N fertilization was used (T9), results were similar to those obtained with the full (100 %) N dose (T2). Plants that received full N fertilization and were leaf spray-inoculated (ILS) at the V2.5 stage with 2.5 doses of inoculant (T14) presented a 26 % increase in root dry weight (RDW) when compared to the non-inoculated 100 % N control (T2). N content (NC) of plants from in-furrow inoculation with a single inoculant dose and 75 % N fertilization (T7) was significantly increased relative to seed inoculation (T3), and similar results were observed for total N in shoot (TNS). On the other hand, NC and TNS of plants from in-furrow inoculation with 2.5 doses of inoculant and 75 % N (T9) were apparently inhibited (Table 4).

**Table 4:** Shoot dry weight (SDW), root dry weight (RDW), N content (NC), total N accumulated in the shoots (TNS), chlorophyll content (CC), root volume (RV), culm diameter (CD), plant height (PH), and MPN (most probable number) of diazotrophic bacteria on leaves, roots and rhizosphere in a greenhouse experiment performed with hybrid maize BRS 3010 in response to different doses of inoculant, levels of N fertilization, and methods of inoculation

Treatment	SDW (g pl^−1^)	RDW (g pl^−1^)	NC (mg g^−1^)	TNS (mg pl^−1^)	CC (µg cm^−2^)	Biometric measurements				Bacterial MPN		
						RV (mL pl^−1^)	CD (mm)	PH (cm)	Leaves (n° cells g^−1^)		Roots (n° cells g^−1^)	Rhizosphere soil (n° cells g^−1^)
T1: C + 75 % N^a^	6.67 c*	3.77 bc	14.74 abc	98.33 cde	12.99^ns^	19.90 abc	9.47^ns^	66.60 bcd	4.57 × 10^6^ cde		1.73 × 10^8^ e	1.55 × 10^6^ b
T2: C + 100 % N	8.01 ab	3.96 bc	14.41 abcd	115.60 abc	13.71	20.30 abc	10.12	73.12 ab	2.92 × 10^6^ de		4.75 × 10^8^ bcde	5.52 × 10^5^ b
T3: SI + 1 dose^b^ + 75 % N	7.43 abc	3.92 bc	11.81 de	87.03 e*	12.93	18.90 bc	9.97	64.40 bcd	2.47 × 10^6^ de		6.24 × 10^8^ abcde	6.12 × 10^5^ b
T4: SI +1 dose + 100 % N	7.56 abc	3.66 c	15.17 ab	113.99 abc	14.76	19.40 bc	9.93	62.10 d	1.14 × 10^7^ bcde		6.24 × 10^8^ abcde	2.12 × 10^6^ b
T5: SI + 2.5 doses + 75 % N	7.71 abc	3.95 bc	14.96 ab	115.93 abc	12.33	20.40 abc	10.08	64.60 bcd	4.31 × 10^6^ cde		6.86 × 10^8^ abcde	5.10 × 10^5^ b
T6: SI + 2.5 doses + 100 % N	8.02 ab	4.47 ab	14.74 abc	117.91 abc	12.99	23.30 a	10.22	66.50 bcd	1.16 × 10^8^ b		2.83 × 10^8^ cde	5.10 × 10^5^ b
T7: IPF + 1 dose + 75 % N	7.29 bc	4.11 bc	15.72 ab	114.39 abc	12.29	18.70 bc	10.08	65.50 bcd	1.22 × 10^8^ b*		5.03 × 10^8^ abcde	3.02 × 10^6^ b
T8: IPF + 1 dose + 100 % N	7.59 abc	3.93 bc	15.96 ab	123.21 a	13.72	19.10 bc	10.58	70.75 abcd	2.70 × 10^7^ bcd		4.58 × 10^8^ abcde	2.52 × 10^7^ a*
T9: IPF + 2.5 doses + 75 % N	8.16 ab	4.07 bc	11.33 e*	91.93 de	12.18	19.60 bc	10.22	65.70 bcd	2.18 × 10^7^ bcd		1.25 × 10^9^ a*	1.52 × 10^6^ b
T10: IPF + 2.5 doses + 100 % N	7.57 abc	3.88 bc	14.66 abcd	109.79 abcd	13.30	18.40 bc	10.07	69.80 abcd	2.40 × 10^9^ a*		3.28 × 10^8^ bcde	8.48 × 10^7^ b
T11: ILS + 1 dose + 75 % N	7.30 abc	3.92 bc	13.62 abcde	99.49 cde	12.28	20.30 abc	10.25	68.80 abcd	2.05 × 10^9^ a*		2.37 × 10^8^ de	1.03 × 10^6^ b
T12: ILS + 1 dose + 100 % N	7.39 abc	4.17 bc	13.48 abcde	98.64 cde	13.38	20.20 abc	10.58	62.87 cd	7.38 × 10^7^ bc		1.02 × 10 abc	4.65 × 10^6^ ab
T13: ILS + 2.5 doses + 75 %N	7.22 bc	4.41 abc	13.47 abcde	96.92 cde	12.38	21.30 ab	10.07	70.20 abcd	7.50 × 10^6^ bcde		9.18 × 10^8^ abcd	1.54 × 10^6^ b
T14: ILS + 2.5 doses + 100 % N	8.13 ab	5.00 a*	13.07 bcde	106.06 abcde	12.63	21.00 abc	10.30	72.80 cd	1.12 × 10^6^ de		7.33 × 10^8^ abcde	3.04 × 10^6^ b
T15: ISS + 1 dose + 75 % N	7.52 abc	3.60 c	13.14 abcde	98.30 cde	13.29	18.10 bc	9.84	68.70abcd	4.07 × 10^6^ cde		4.84 × 10^8^ cde	1.34 × 10^6^ b
T16: ISS + 1 dose + 100 % N	7.92 abc	4.38 abc	16.05 a	126.731 a	13.97	20.50 abc	10.07	75.70a	1.10 × 10^6^ de		9.94 × 10^8^ abc	1.40 × 10^6^ b
T17: ISS + 2.5 doses + 75 % N	8.50 a	4.47 ab	11.95 cde	101.15 bcde	11.80	21.30 ab	10.43	73.10 ab	1.11 × 10^6^ de		3.66 × 10^8^ cde	1.50 × 10^6^ b
T18: ISS + 2.5 doses + 100 % N	7.72 abc	3.88 bc	15.71 ab	121.13 ab	13.91	17.70 c	10.21	71.40 abc	6.25 × 10^5^ e		1.14 × 10^9^ ab	1.65 × 10^6^ b
*p* value	0.0058	0.015	<0.0001	<0.0001	0.5525	0.00492	0.6696	0.0127	<0.0001		0.0192	0.0002
CV (%)	8.08	12.81	10.57	10.06	13.92	12.02	6.38	10.6	11.2		6.65	9.24

Parameters determined 52 days after seedling emergence

Means (five replicates) followed by the same letter on the same column are not significantly different from one another according to Duncan’s test (*p* ≤ 0.05), whereas means followed by * are significantly different from treatment 2 (T2) according to Dunnet’s test (*p* ≤ 0.05). *ns* not significant

*C* non-inoculated control, *SI* standard seed inoculation, *IPF* inoculation at planting in the furrow, *ILS* inoculation by leaf spray at the V2.5 stage, *ISS* inoculation by soil spray at the V2.5 stage

^a^N: 75 % (90 kg ha^−1^) and 100 % (120 kg ha^−1^), split in equal amounts every 8 days

^b^Inoculant dose:1× (1.0 × 10^5^ cells seeds^−1^ or 1.0 × 10^5^ cells plant^−1^)

Chlorophyll content (CC) and culm diameter (CD) were not affected by any of the treatments studied (Table 4). Root growth, as indicated by root volume (RV), responded positively to seed inoculation (SI) with 2.5 doses of inoculant and full N fertilization (T6) when compared to all treatments of inoculation in-furrow (T7–T10). Inoculation by soil spray with one dose of inoculant in addition to full N fertilization (T16) significantly increased plant height (PH) relative to all treatments with seed inoculation and to the non-inoculated control that received 75 % N (Table 4).

Internal leaf colonization by diazotrophs, as estimated by the most-probable number (MPN) technique, was significantly superior in plants from treatments 6, 7, 10, and 11, when compared to plants from the non-inoculated controls (T1 and T2) (Table 4). Higher internal leaf populations of diazotrophic bacteria were observed in plants from treatments in which one dose of inoculant was applied by leaf spraying at the V2.5 stage (T11 and T12), but also with 2.5 doses of inoculation in-furrow with 100 % of N (T10). Significantly increased internal root colonization by diazotrophic bacteria was observed in plants from T9, where 2.5 doses of inoculant were applied in-furrow with 75 % of N, in comparison to plants from the non-inoculated controls (T1 and T2). When rhizospheric soil was analyzed, the largest bacterial populations were observed in association with T8, which received a single dose of inoculant in-furrow and 100 % N (Table 4).

### Greenhouse experiment with wheat

No significant differences were observed in SDW, but RDW varied significantly among treatments (Table 5). Application of one dose of inoculant by foliar spray at the tiller stage 3 (ILS) with 100 % N (T12) exhibited a 57 % increase when compared with the non-inoculated 100 % N treatment. ILS and soil spray inoculation at the tiller stage 3 (ISS) with 2.5 doses of inoculant and added of 100 % N (T14 and T18) promoted highest N contents (NC) and T14 also resulted in the highest accumulation of N in the shoots (TNS). Chlorophyll contents (CC) were significantly superior in the inoculated treatments that received reduced N fertilization, regardless of how inoculation was performed, except for conventional seed inoculation, when compared to the non-inoculated control with fertilization with either 75 or 100 % N. The application of 2.5 doses of inoculant in-furrow, combined with full N fertilization resulted in the highest CC among the treatments. No significant differences in RV were observed. Plants that received 100 % N and were inoculated by leaf spray (T12) presented the largest number of tillers, and it is worth mentioning that in-furrow application of a single inoculant dose combined with 75 % N (T7) was superior to the non-inoculated 75 % N treatment (T1) (Table 5).

**Table 5:** Shoot dry weight (SDW), root dry weight (RDW), N content (NC), total N accumulated in the shoots (TNS), chlorophyll content (CC), root volume (RV), number of tillers (NT), and MPN (most probable number) of diazotrophic bacteria on leaves, roots and rhizosphere soil in a greenhouse experiment with wheat cultivar the BRS Gaivota in response to different doses of inoculant, levels of N fertilization, and methods of inoculation

Treatment	SDW (g pl^−1^)	RDW (g pl^−1^)	NC (mg g^−1^)	TNS (mg pl^−1^)	CC (µg cm^−2^)	Biometric measurements		Microbiological parameters		
						RV (mL pl^−1^)	NT (n° pl^−1^)	Leaf (n° cells g^−1^)	Root (n° cells g^−1^)	Rhizosphere soil (n° cells g^−1^)
T1: C + 75 %N^a^	1.64^ns^	1.71 bc	22.40 d	36.72 b	17.90 b	9.0^ns^	4.60 c	1.26 × 10^5^ cd	2.64 × 10^8^ abcd	3.30 × 10^6^ bcd
T2: C + 100 %N	1.76	1.66 bc	27.42 abc	48.16 ab	17.89 b	9.1	4.90 bc	4.07 × 10^4^ d	7.92 × 10^8^ abcd	5.33 × 10^6^ d
T3: SI + 1 dose^b^ + 75 %N	1.78	1.98 abc	26.79 abcd	47.98 ab	19.08 ab	8.1	5.00 bc	8.94 × 10^5^ cd	5.28 × 10^8^ abcd	2.24 × 10^6^ bcd
T4: SI +1 dose +100 %N	1.80	2.23 abc	24.41 abcd	46.42 ab	19.67 ab	11.1	6.20 ab	6.95 × 10^5^ bcd	9.00 × 10^7^ bcd	2.19 × 10^6^ cd
T5: SI + 2.5 doses + 75 %N	1.62	1.96 abc	27.25 abc	44.06 ab	19.34 ab	7.4	5.00 bc	3.98 × 10^5^ cd	4.97 × 10^8^ abcd	1.00 × 10^7^ abcd
T6: SI + 2.5 doses + 100 %N	1.83	2.35 ab	25.65 abcd	47.07 ab	19.15 ab	9.6	5.50 abc	3.69 × 10^6^ abc	6.26 × 10^7^ cd	4.14 × 10^6^ abcd
T7: IPF + 1 dose + 75 %N	1.79	2.22 abc	24.54 abcd	44.06 ab	21.34 a	9.2	6.20 ab	5.04 × 10^6^ abc*	5.42 × 10^8^ abcd	1.38 × 10^7^ abcd
T8: IPF + 1 dose + 100 %N	1.68	1.98 abc	24.91 abcd	41.90 ab	21.07 ab	9.3	5.20 abc	1.07 × 10^6^ abcd	1.60 × 10^8^ d	4.46 × 10^6^ abcd
T9: IPF + 2.5 doses + 75 %N	1.56	1.81 bc	25.70 abcd	40.15 ab	19.58 ab	7.8	4.40 c	2.09 × 10^6^ abcd	1.03 × 10^9^ ab	3.32 × 10^7^ abc
T10: IPF + 2.5 doses + 100 %N	1.64	1.93 abc	27.05 abc	44.42 ab	22.36 a*	9.2	5.00 bc	1.14 × 10^6^ abcd	4.84 × 10^8^ abcd	3.29 × 10^7^ a*
T11: ILS + 1 dose + 75 %N	1.65	2.33 ab	23.60 bcd	38.60 b	19.63 ab	9.8	5.90 abc	2.40 × 10^7^ a*	4.17 × 10^8^ abcd	1.12 × 10^8^ ab
T12: ILS + 1 dose + 100 %N	1.66	2.61 a*	24.42 abcd	40.38 ab	20. 49 ab	12.1	6.60 a	2.74 × 10^6^ abc	8.94 × 10^7^ bcd	2.98 × 10^6^ cd
T13: ILS + 2.5 doses + 75 %N	1.50	1.57 c	26.23 abcd	38.46 b	21.99 a*	9.6	4.60 c	7.06 × 10^5^ abcd	5.17 × 10^8^ abcd	3.95 × 10^6^ bcd
T14: ILS + 2.5 doses + 100 %N	1.84	2.15 abc	28.48 a	51.64 a	20.35 ab	8.0	5.70 abc	3.82 × 10^7^ a*	6.82 × 10^7^ bcd	6.20 × 10^6^ abcd
T15: ISS + 1 dose + 75 %N	1.62	1.76 bc	23.17 cd	37.29 b	21.26 a	8.4	5.00 bc	9.72 × 10^6^ ab*	9.92 × 10^8^ abc	5.36 × 10^6^ bcd
T16: ISS + 1 dose + 100 %N	1.72	1.55 c	27.87 ab	47.11 ab	16.65 ab	6.4	5.90 abc	1.04 × 10^6^ abc	2.40 × 10^9^ a*	2.42 × 10^6^ cd
T17: ISS + 2.5 doses + 75 % N	1.54	1.94 abc	27.25 abc	41.46 ab	19.56 ab	8.4	5.00 bc	1.45 × 10^6^ abc	8.23 × 10^8^ abcd	2.13 × 10^6^ cd
T18: ISS + 2.5 doses + 100 % N	1.75	2.09 abc	28.88 a	43.82 ab	20.01 ab	10.3	6.30 ab	1.90 × 10^6^ abc	1.21 × 10^9^ abcd	1.77 × 10^6^ d
*p* value	0.5401	0.0497	0.0217	0.0043	0.0015	0.2877	0.0125	<0.0001	0.0002	0.0134
CV (%)	21.31	24.74	11.44	21.06	13.04	30.25	18.74	14.32	7.5	8.09

Parameters determined at 54 days after seedling emergence

Means (five replicates) followed by the same letter on the same column are not significantly different from one another according to Duncan’s test (*p* ≤ 0.05), whereas means followed by * are significantly different from treatment 2 (T2) according to Dunnet’s test (*p* ≤ 0.05). *ns* not significant

*C* non-inoculated control, *SI* standard seed inoculation, *IPF* inoculation at planting in the furrow, *ILS* inoculation by leaf spray at the tiller stage 3, *ISS* inoculation by soil spray at the at the tiller stage 3

^a^N: 75 % (60 kg ha^−1^) and 100 % (80 kg ha^−1^), split in equal amounts every 8 days

^b^Inoculant dose:1× (1.74 × 10^4^ cells seed^−1^ or 1.74 × 10^4^ cells plant^−1^)

Bacterial populations were significantly larger on the leaves (internal) of plants from treatments with foliar spray inoculation (T11 and T14), when compared to non-inoculated treatments (T1 and T2) (Table 5). In the case of the roots, diazotrophic bacteria were more numerous when a single dose of inoculant was sprayed on the soil with full N fertilization (T16), whereas for the rhizosphere significantly larger bacterial populations were observed for in-furrow application of 2.5 doses of inoculant (T10), compared to non-inoculated controls (T1 and T2) (Table 5).

These preliminary greenhouse experiments were performed to verify possible effects on plant growth and colonization of diazotrophic bacteria, aiming to obtain an indication of the treatments that could be taken to the field.

### Field trials with maize and wheat

For the summer crop (maize), in Cachoeira Dourada, highest SDW was observed when seeds received a single dose of inoculant and 75 % N (T5) (Table 6). No significant differences among treatments were observed in Luiz Eduardo Magalhães for SDW, but when NC was considered inoculation by foliar spray with two doses of inoculant and 75 % of N (T8) was significantly superior to the 100 % N (T2) control treatment. No significant effects of any treatment were observed for SDW of the plants from Ponta Grossa. Inhibitory effects of the soil spray inoculation with two doses of inoculant (T10) on NC were evident both in Ponta Grossa and Cachoeira Dourada (Table 6).

**Table 6:** Shoot dry weight (SDW) and N content (NC) in maize plants from field experiments performed in three different regions of Brazil (Cachoeira Dourada, hybrid 2B707 HX, Luis Eduardo Magalhães, hybrid 2B707 HX, and Ponta Grossa, hybrid P4285 H), in response to different doses of N fertilizer, doses of inoculant and methods of inoculation with *Azospirillum brasilens*

Treatment	Cachoeira Dourada		Luis E. Magalhães		Ponta Grossa	
	SDW (g pl^−1^)	NC (mg g^−1^)	SDW (g pl^−1^)	NC (mg g^−1^)	SDW (g pl^−1^)	NC (mg g^−1^)
T1: C	29.45 c	23.08 a	68.82^ns^	30.08 b	49.08^ns^	18.91 d
T2: C + 100 % N^a^	36.01 abc	22.19 a	66.30	30.05 b	53.50	23.69 ab
T3: C + 75 % N	33.01 ab	21.16 ab	65.78	30.97 ab	56.09	23.74 ab
T4: SI + 1 dose^b^ + 100 % N	36.68 abc	20.93 ab	71.34	31.60 ab	54.12	24.67 a
T5: SI + 1 dose + 75 % N	41.13 a	21.60 ab	71.26	33.43 ab	58.33	23.86 ab
T6: IPF + 2 doses + 75 % N	34.41 abc	20.31 abc	70.50	32.79 ab	53.95	21.11 c
T7: IPF + 4 doses + 75 % N	34.23 abc	19.18 bc	65.45	34.10 ab	54.14	22.15 bc
T8: ILS + 2 doses + 75 % N	34.26 abc	19.07 bc	69.75	35.31 a	49.48	22.71 bc
T9: ILS + 4 doses + 75 % N	36.34 abc	18.68 bc	77.63	33.88 ab	54.97	20.94 c
T10: ISS + 2 doses + 75 % N	39.47 ab	17.69 c	71.94	30.58 b	50.60	20.82 c
T11: ISS + 4 doses + 75 % N	32.18 bc	20.76 ab	75.41	31.36 ab	47.73	20.96 c
*p* value	0.0438	0.0028	0.5303	0.0764	0.4597	<0.0001
CV (%)	34.64	10.82	13.97	10.07	15.15	6.77

Plants harvested at 30, 56 and 61 days after emergence, in Cachoeira Dourada, Luis Eduardo Magalhães and Ponta Grossa, respectively

Means (six replicates) followed by the same letter on the column are not significantly different from one another according to Duncan’s test (*p* ≤ 0.05). *ns* not significant

*C* non-inoculated control, *SI* standard seed inoculation at sowing, *IPF* inoculation at planting in the furrow, *ILS* inoculation by leaf spray at the V2.5 stage, *ISS* inoculation by soil spray at the V2.5 stage

^a^N, 75 % (24 kg ha^−1^ at sowing + 67.5 kg ha^−1^ as side dress); 100 % (24 kg ha^−1^ at sowing + 90 kg ha^−1^ as side dress)

^b^Dose, 1 dose (1.0 × 10^5^ cells seeds^−1^ or 1.0 × 10^5^ cells plant^−1^)

**Fig. 1:**
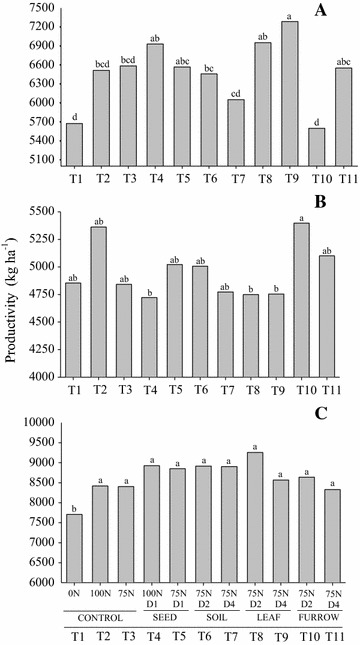
Grain yield (kg ha^−1^) of maize in location of Cachoeira Dourada (**A** hybrid 2B707 HX), Luis Eduardo Magalhães (**B** hybrid 2B707 HX), and Ponta Grossa (**C** hybrid P4285 H), in response to different managements with N-fertilizer and inoculants with *Azospirillum brasilense*. All treatments received 24 kg of N ha^−1^ at sowing and N-treatments refers to the complementary N-fertilization applied at 35 days after emergence: 0 N (no N fertilizer); 100 N (100 % N); 75 N (25 % reduction in the complementary N fertilization). Inoculant doses: D1 (one standard dose, 1.0 × 10^5^ cells seeds^−1^ or 1.0 × 10^5^ cells plant^−1^); D2 (2 doses); D4 (4 doses). Inoculation methods: CONTROL (non-inoculated); SEED (standard seed inoculation); SOIL (soil spray inoculation at the V2.5 stage); FOLIAR (leaf spray inoculation at the V2.5 stage); FURROW (inoculation in the planting furrow at sowing). Yields were obtained from grains harvested in the central lanes of each replicate (8 m^2^), dried to 13 % of moisture and estimated to 1 ha. Means of six replicates and different letters indicate statistical difference (Duncan’s test at *p* ≤ 0.05)

Leaf spray inoculation at V2.5 stage with four (T9) and two (T8) doses of inoculant and 75 % of N promoted yield increases of 773 and 439 kg ha^−1^ over non-inoculated plants which received full N fertilization (T2) in Cachoeira Dourada (Fig. 1A). In Luiz Eduardo Magalhães, although not significantly different, plants receiving two doses of inoculant in-furrow (T10) with 75 %N had yield increases of 555 kg ha^−1^ relative to the 75 % N control (T3; Fig. 1B). Still in Luiz Eduardo Magalhães, inoculation in-furrow (T10) was significantly superior to treatments with foliar spray inoculation (T8, T9) and seed inoculation with a single inoculant dose receiving full N fertilization (T4) (Fig. 1B). In Ponta Grossa, even though no significant differences in grain yield could be observed, inoculation by foliar spray with two inoculant doses (T8) increased yield by 850 kg ha^−1^ when compared to the 100 % N control (T2) (Fig. 1C).

**Fig. 2:**
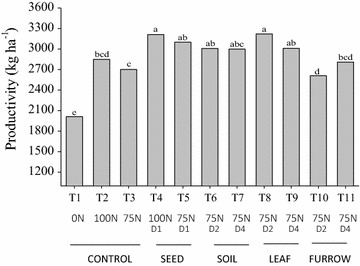
Grain yield (kg ha^−1^) of wheat cultivar BRS Pardela in Ponta Grossa, PR, in response to different managements with N-fertilizer and inoculants with *Azospirillum brasilense*. All treatments received 24 kg of N ha^−1^ at sowing and N-treatments referred to the complementary N-fertilization applied at 35 days after emergence: 0 N (no N fertilizer); 100 N (100 % N); 75 N (25 % reduction in the complementary N fertilization). Inoculation treatments consisted of SI (standard seed inoculation at sowing), IPF (inoculation in the furrow at sowing), ILS (inoculation by leaf spray at the tiller stage 3), ISS (inoculation by soil spray at the tiller stage 3), with one D1 (1.74 × 10^4^ cells seed^−1^ or 1.74 × 10^4^ cells plant^−1^); D2 or D4 doses. Yields were obtained from grains harvested in the central lanes of each replicate (7.2 m^2^), dried to 13 % of moisture and estimated to 1 ha. Means of six replicates and different letters indicate statistical difference (Duncan’s test at *p* ≤ 0.05)

In the comparison of treatments and the control receiving 75 % of N, wheat yield responded significantly to inoculation of seeds, and leaf and soil spray, confirming the benefits of *Azospirillum* inoculation (Fig. 2). The best results were obtained when two doses of inoculants were employed as leaf spray at the tiller stage 3 (Fig. 2).

## Discussion

Recent data indicate that about 25 million doses of *Bradyrhizobium* spp. inoculants for soybeans, and 2 million doses of *A. brasilense* inoculants, for maize and wheat, are sold annually in Brazil (Marks et al. 2013). Although the commercialization of products containing *Azospirillum* seems proportionally low, such inoculants only reached the Brazilian market about a half decade ago, while rhizobia have been in the market for over 60 years. In addition, very few field studies have been conducted under Brazilian conditions, and no options to avoid the incompatibility between the bacteria and the array of seed treatment agrichemicals are available. The results reported here are largely applicable to other important producing countries of South America and Africa.

Full replacement of N fertilizers for grasses by *A. brasilense* may not be feasible, because of the modest contribution of biological nitrogen fixation by the bacterium. However, the combination of all minor contributions by *Azospirillum* to plant growth may result in plants that are more efficient to absorb water and nutrients from soil, thus enhancing plant nutrition and growth (Stancheva et al. 1992; Dobbelaere et al. 2001, 2002; Bashan et al. 2004; Bashan and de-Bashan 2010; Hungria et al. 2010, 2013, 2015; Kouchebagh et al. 2012).

In our study, we observed that spray inoculation, either on the leaves or on the soil surface increased maize plant growth. In general, when inoculation with *Azospirillum* was associated to 75 % of the complementary dose of N, plant growth was superior to non-inoculated plants receiving 100 % N. Therefore, the replacement of 25 % of the N-fertilizer by *Azospirillum* is profitable for the farmer and the environment.

The efficiency of *Azospirillum* spp. may be negatively affected by the presence of high levels of N fertilizers, due to the rapid decrease in the activity of nitrogenase (Hartmann 1989), and in general such negative effects were also observed in our study. On the other hand, stimulation in response to the association between lower doses of N and *Azospirillum* are reported (Piccinin et al. 2013). For example, in our greenhouse experiment with wheat, shoot dry matter was not affected by inoculation, but foliar spray inoculation resulted in increased root systems and N accumulation in the shoots. Reports from literature show increases in root growth and N accumulation in the shoots of maize and *Setaria* grass inoculated with *A. brasilense* (Cohen et al. 1980), effect that has been attributed to morphological and physiological changes in the roots, promoting water and nutrient uptake by the plants (Dobbelaere et al. 2001, 2002).

Alterations in the root system are probably caused by the presence of plant growth hormones, especially indoleacetic acid (IAA) produced and secreted by *Azospirillum*, thus playing a major role in plant growth promotion (Bashan and Holguin 1997). In addition to IAA production, *Azospirillum*, as well as other PGPB have been implicated with an array of mechanisms that act simultaneously or sequentially and result in increased plant growth (Bashan and de-Bashan 2010), root formation, cell division and growth, and production of lateral and adventitious roots (Werner et al. 2003; Bhattacharyya and Jha 2012). *Azospirillum* has also been implicated with higher photosynthesis rates and photosynthetic pigments (Bashan et al. 2006; Barassi et al. 2008; Hungria et al. 2010).

In contrast to the satisfactory results of foliar spray inoculation that we observed in this study, in-furrow inoculation with elevated doses of *Azospirillum* was somewhat inhibitory to plant growth. Dobbelaere et al. (2002) suggested that high concentrations of plant hormones, which are stimulatory at low concentrations, may have negative effects on plant growth. The higher abundance of *Azospirillum* in the root environment may have increased the secretion of such hormones, thus inhibiting root growth. Similar negative effects of high concentrations of *Azospirillum* have been previously reported for wheat (Bashan 1986) and maize (Fallik et al. 1988). Hungria et al. (2013) observed benefits only at lower doses of inoculation, and growth inhibition with higher doses of *Azospirillum* when soybean and common bean were co-inoculated with rhizobia and the same strains of *Azospirillum* employed in our study. Inoculation with high concentrations of *Azospirillum* also decreased the N content of field-grown plants in Cachoeira Dourada and Ponta Grossa, but not in Luiz Eduardo Magalhães. Our results present strong evidence that field inoculation with PGPB must pay attention to inoculant doses, and point to the need of fine adjustments so as not to cross the threshold of growth stimulation and inhibition.

In this paper, we report positive effects of inoculation on maize root volume, in addition to increases in plant height when inoculant was sprayed on the soil, whereas foliar spray inoculation resulted in more tillers in wheat, probably related to plant growth hormones that might have been present in the inoculants or were produced by the bacteria. *Azospirillum* colonizes plant niches that are protected from oxygen and, as a result, nitrogenase is maintained functional (Dobbelaere et al. 2003). Colonization of intercellular spaces between the epidermis and the cortex, and of the outermost layers of the cortex of inoculated roots is frequently observed (Patriquin et al. 1983; Mostajeran et al. 2007). *A. brasilense* was predominantly located between the apoplast and the epidermal cells of wheat roots (Nabti et al. 2010). However, these bacteria can also colonize leaves (Bashan 1998). Even after surface disinfection, those authors observed that the bacteria were more frequent on the roots, followed by culms and leaves of maize plants. In this study, we observed higher numbers of bacteria on roots, but they were also recovered, although in 100-fold lower populations, from leaves.

*Azospirillum* survives well in Brazilian soils and can be found in association with plants even when no inoculation is done. For example, Pereyra et al. (2010) detected populations of *A. brasilense* of 3 × 10^3^ colony forming units (CFU) g^−1^ of roots of non-inoculated cucumber (*Cucumis sativus*) plants, whereas the roots of inoculated plants contained as much as 8 × 10^6^ CFU g^−1^. In our study, both maize and wheat plants showed improved rhizosphere and root colonization by diazotrophic bacteria in some treatments that received inoculation in-furrow. Bacterial abundances also showed some increase when non-inoculated treatments were considered, but responses were more expressive when inoculation was performed.

Inoculation by soil spraying resulted in increased internal colonization of aerial plant parts of wheat by *Azospirillum*, indicating high bacterial mobility through the plants. Several authors (Baldani et al. 1992; James et al. 1994; Souza et al. 2004) have suggested that the presence of diazotrophic bacteria in xylem vessels may indicate that this may be one route of bacterial migration to different plant parts. The internal colonization of both maize and wheat leaves also increased in response to foliar spray inoculation, suggesting that in this case the stomata acted as a passive doorway for bacteria, since for leaf sprays the soil was covered to avoid cross contamination and root colonization by inoculant bacteria. In fact, in a scan electron microscopy (SEM) study performed by Baldotto et al. (2011) to evaluate the colonization of pineapple [*Ananas comosus* (L.) Merril] by *Herbaspirillum seropedicae*, bacterial aggregates could be observed over trichomes and junctions of the epidermis cell walls, as well as on the external periclinal wall and near stomata complexes. Those authors suggested that their observations evidenced that bacterial penetration in the leaves occurred passively via stomata, and colonization began in the sub-stomata chamber and progressed on through the intercellular spaces of the spongy chlorenchyma of the leaf mesophyll. Similarly, Souza et al. (2004) have observed that bacterial distribution throughout maize leaves takes place via colonization of the epidermal cells of the adaxial face, with the formation of aggregates on the epidermis or near the stomata. Another evidence of leaf colonization obtained in our study is that our tests have indicate that *Azospirillum* can barely survive 2 h on the leaves, and far less under field conditions (data not shown), giving more support to the hypothesis that the increase in bacteria inside the leaves could be related to colonization associated with leaf spray. It is worth reinforcing that in our field experiments with maize in general yields of inoculated plants with maize and receiving 75 % of the complementary dose of N-fertilizer produced as much as non-inoculated plantas receiving 100 % of N.

Inoculation with *A. brasilense* may result in increases of wheat yield, or maintain actual yield standards but with a reduction in the amounts of N fertilizers applied (Rothballer et al. 2003; Hungria et al. 2010; Venieraki et al. 2011; Piccinin et al. 2013). Increased yield in wheat is generally attributed to an increase in the number of fertile tillers (Salantur et al. 2006), and inoculation with *A. brasilense* resulted in more tillers in wheat plants grown in disinfected soil (Saubidet et al. 2002). In our study, inoculation by both soil and leaf sprays also resulted in more tillers in the greenhouse experiment, strengthening the hypothesis of increased tillering as a mechanism of yield promotion in response to inoculation with *Azospirillum*. Although tillering was not evaluated in the field, our data confirmed the benefits of *A. brasilense* to wheat grain yield, since all treatments that received inoculants produced more grains than the non-inoculated controls, even when full N fertilization was performed. In addition, in our field trial with wheat in general the presence of the inoculant applied to the seeds, or by soil or leaf spray allowed a 25 % reduction in the rates of N fertilization with better yields than when 100 % N was employed without inoculation.

Grain yield of inoculated maize increased due to improved N nutrition, and part could be attributed to biological nitrogen fixation by *Azospirillum* spp. (Dobbelaere et al. 2003), but the magnitude of the response depended on the level of N fertilization practiced, as reported before (Piccinin et al. 2013). In studies of maize seed inoculation with different species (*A. lipoferum* and *A. brasilense*) and strains (including the two strains of the present study), Hungria et al. (2010) observed yield increases of up to 30 % (or 823 kg ha^−1^), which were attributed to improved N nutrition and to increased nutrient absorption by inoculated plants with larger root systems. In Cerrado region of Brazil, 29 % yield increases in maize were due to inoculation with *Azospirillum* (Ferreira et al. 2013).

In Brazil it is estimated that 70 % of the N fertilizers are imported from other countries, resulting in high costs for agricultural activities (Hungria et al. 2013). The increased yields obtained from treatments that receive adequate doses of inoculants, combined with a 25 % reduction in urea application present an attractive alternative to reduce costs in agriculture. For example, in the experiment performed in Cachoeira Dourada, inoculation by leaf spray with the highest inoculant dose promoted an increase of 773 kg ha^−1^ in grain yield of maize over the treatment that received the full dose of N fertilizer (100 % N), with no inoculation.

In conclusion, taking into account the search for more conservative agricultural systems, inoculation with *A. brasilense* stands as a promising strategy to contribute to increased sustainability. However, in times when more and more pesticides for seed treatment are released, and so little is known about their toxicity to inoculated *Azospirillum* bacteria, compatibility with inoculants applied to the seed can seriously limit microbial contribution. In our study we have identified alternative methods of inoculation to avoid the contact of *Azospirillum* with pesticides applied to the seeds, with an emphasis on leaf spray at the beginning of the vegetative phase. Alternative methods of inoculation may increase the utilization of such bacteria in the field and help reduce agricultural costs.

## References

[CR1] Baldani VLD, Baldani JI, Olivares FL, Döbereiner J (1992). Identification and ecology of *Herbaspirillum seropedicae* and the closely related *Pseudomonas rubrisalbicans*. Symbiosis.

[CR2] Baldotto LEB, Olivares FL, Bressan-Smith R (2011). Structural interaction between gfp-labeled diazotrophic endophytic bacterium *Herbaspirillum seropedicae* ram10 and pineapple plantlets ‘Vitória’. Braz J Microbiol.

[CR3] Barassi CA, Sueldo RJ, Creus CM, Carrozzi LE, Casanovas WM, Pereyra MA. Potencialidad de *Azospirillum* en optimizar el crecimiento vegetal bajo condiciones adversas. In: Cassán FD, Garcia de Salamone I, editor. *Azospirillum* sp.: cell physiology, plant interactions and agronomic research in Argentina. Argentina: Asociación Argentina de Microbiologia, Buenos Aires. 2008. pp 49–59.

[CR4] Bashan Y (1986). Significance of timing and level of inoculation with rhizosphere bacteria on wheat plants. Soil Biol Biochem.

[CR5] Bashan Y (1998). *Azospirillum* plant growth-promoting strains are nonpathogenic on tomato, pepper, cotton, and wheat. Can J Microbiol.

[CR6] Bashan Y, Bustillos JJ, Leyva LA, Hernadez J-P, Bacilio M (2006). Increase in auxiliary photoprotective photosynthetic pigments in wheat seedlings induced by Azospirillum brasilense. Biol Fertil Soils.

[CR7] Bashan Y, de-Bashan LE, Hillel D (2005). Plant growth-promoting. Encyclopedia of soils in the environment.

[CR8] Bashan Y, de-Bashan LE (2010). How the plant growth-promoting bacterium *Azospirillum* promotes plant growth—a critical assessment. Adv Agron.

[CR9] Bashan Y, de-Bashan LE, Prabhu SR, Hernandez J-P (2014). Advances in plant growth-promoting bacterial inoculant technology: formulation and practical perspectives (1998–2013). Plant Soil.

[CR10] Bashan Y, Holguin G (1997). *Azospirillum*-plant relationship: environmental and physiological advances (1990–1996). Can J Microbiol.

[CR11] Bashan Y, Holguin G, de-Bashan LE (2004). *Azospirillum*-plant relationships: physiological, molecular, agricultural and environmental advances. Can J Microbiol.

[CR12] Bhattacharyya PN, Jha DK (2012). Plant growth-promoting bacteria (PGPB): emergence in agriculture. World J Microbiol Biotechnol.

[CR13] Bottini R, Fulchieri M, Pearce D, Pharis R (1989). Identification of gibberelins A1, A3, and iso-A3 in cultures of *A. lipoferum*. Plant Physiol.

[CR14] Campo RJ, Araujo RS, Hungria M (2009). Nitrogen fixation with the soybean crop in Brazil: compatibility between sedd treatment with fungicides and bradyrhizobial inoculants. Symbiosis.

[CR15] Cohen E, Okon Y, Kigel J, Nur I, Henis Y (1980). Increase in dry weight and total nitrogen content in *Zea mays* and *Setaria italica* associated with nitrogen-fixing *Azospirillum* spp. Plant Physiol.

[CR16] de-Bashan LE, Hernandez J-P, Bashan Y (2012). The potential contribution of plant growth-promoting bacteria to reduce environmental degradation—a comprehensive evaluation. Appl Soil Ecol.

[CR17] Díaz-Zorita M, Fernández-Canigia MV (2009). Field performance of a liquid formulation of *Azospirillum brasilense* on dryland wheat production. Eur J Soil Biol.

[CR18] Dobbelaere S, Croonenborghs A, Thys A, Ptacek D, Okon Y, Vanderleyden J (2002). Effect of inoculation with wild type *Azospirillum brasilense* and *A. irakense* strains on development and nitrogen uptake of spring wheat and grain maize. Biol Fertil Soils.

[CR19] Dobbelaere S, Croonenborghs A, Thys A, Ptacek D, Vanderleyden J, Dutto P, Labandera-Gonzalez C, Caballero-Mellado J, Aguirre JF, Kapulnik Y, Brener S, Burdman S, Kadouri D, Sarig S, Okon Y (2001). Responses of agronomically important crops to inoculation with *Azospirillum*. Aust J Plant Physiol.

[CR20] Dobbelaere S, Vanderleyden J, Okon Y (2003). Plant growth-promoting effects of diazotrophs in the rhizosphere. Crit Rev Plant Sci.

[CR21] Döbereiner J, Baldani VLD, Baldani JI (1995). Como isolar e identificar bactérias diazotróficas de plantas não-leguminosas.

[CR22] Elslahi RH, Osman AG, Sherif AM, Elhussein AA (2014). Comparative study of the fungicide Benomyl toxicity on some plant growth promoting bacteria and fungi in pure cultures. Interdiscip Toxicol..

[CR23] Embrapa (2006). Sistema brasileiro de classificação de solos.

[CR24] Fallik E, Okon Y, Fischer M (1988). Growth response of maize roots to *Azospirillum* inoculation: effect of soil organic matter content, number of rhizosphere bacteria and timing of inoculation. Soil Biol Biochem.

[CR25] Ferreira AS, Pires RR, Rabelo PG, Oliveira RC, Luz JMQ, Brito CH (2013). Implications of *Azospirillum brasilense* inoculation and nutrient addition on maize in soils of the Brazilian Cerrado under greenhouse and field conditions. Appl Soil Ecol.

[CR26] Hartmann A (1989). Ecophysiological aspects of growth and nitrogen fixation in *Azospirillum* spp. Plant Soil.

[CR27] Hickman JS, Shroyer JP (1994). Corn production handbook.

[CR28] Hungria M, Araujo RS. Manual de métodos empregados em estudos de microbiologia agrícola. EMBRAPA-SPI, Brasília, Brazil. 1994. pp. 542 (ISSN 0101-9716).

[CR29] Hungria M, Campo RJ, Mendes IC, Graham PH, Singh RP, Shankar N, Jaiwal PK (2006). Contribution of biological nitrogen fixation to the N nutrition of grain crops in the tropics: the success of soybean (*Glycine max* L. Merr.) in South America. Nitrogen nutrition and sustainable plant productivity.

[CR30] Hungria M, Campo RJ, Souza EM, Pedrosa FO (2010). Inoculation with selected strains of *Azospirillum brasilense* and *A. lipoferum* improves yields of maize and wheat in Brazil. Plant Soil.

[CR31] Hungria M, Loureiro MF, Mendes IC, Campo RJ, Graham PH, Werner W, Newton WE (2005). Inoculant preparation, production and application. Nitrogen fixation in agriculture, forestry, ecology and the environment.

[CR32] Hungria M, Nogueira MA, Araujo RS (2013). Co-inoculation of soybeans and common beans with rhizobia and azospirilla: strategies to improve sustainability. Biol Fertil Soils.

[CR33] Hungria M, Nogueira MA, Araujo RS (2015). Soybean seed co-inoculation with *Bradyrhizobium* spp. and *Azospirillum brasilense*: a new biotechnological tool to improve yield and sustainability. Am J Plant Sci.

[CR34] James EK, Reis VM, Olivares FL, Baldani JI, Döbereiner J (1994). Infection of sugar cane by the nitrogen fixing bacterium *Acetobacter diazotrophicus*. J Exp Bot.

[CR35] Kaschuk G, Hungria M, Leffelaar PA, Giller KE, Kuyper TW (2009). Differences in photosynthetic behavior and leaf senescence of soybean (*Glycine max* [L.] Merrill) dependent on N_2_ fixation or nitrate supply. Plant Biol.

[CR36] Klute A (1986). Methods of soil analysis, part 1, physical and mineralogical methods.

[CR37] Kouchebagh SB, Mirshekari B, Farahvash F (2012). Improvement of corn yield by seed biofertilization and urea application. World Appl Sci J.

[CR38] Large EC (1954). Growth stages in cereals illustration of the Feeks scales. Plant Pathol.

[CR39] Marks BB, Megías M, Nogueira MA, Hungria M (2013). Biotechnological potential of rhizobial metabolites to enhance the performance of *Bradyrhizobium* spp. and *A. brasilense* inoculants with soybean and maize. ABM Express.

[CR40] Mohiuddin M, Mohammed MK (2013). Influence of fungicide (Carbendazim) and herbicides (2,4-D and Metribuzin) on non-target beneficial soil microorganisms of Rhizospheric Soil of Tomato Crop. IOSR J Environ Sci Toxicol Food Technol.

[CR41] Mostajeran A, Amooaghaie R, Emtiazi G (2007). The participation of the cell wall hydrolytic enzymes in the initial colonization of *Azospirillum brasilense* on wheat roots. Plant Soil.

[CR42] Moutia JFY, Saumtally S, Spaepen S, Vanderleyden J (2010). Plant growth promotion by *Azospirillum* sp. in sugarcane is influenced by genotype and drought stress. Plant Soil.

[CR43] Nabti E, Sahnoune M, Ghoul M, Fischer D, Hofmann A, Rothballer M, Schmid M, Hartmann A (2010). Restoration of growth of durum wheat (*Triticum durum* var. waha) under saline conditions due to inoculation with the rhizosphere bacterium *Azospirillum brasilense* NH and extracts of the marine alga *Ulva lactuca*. J Plant Growth Regul.

[CR44] Okon Y, Labandera-Gonzalez C (1994). Agronomic applications of *Azospirillum*: an evaluation of 20 years worldwide field inoculation. Soil Biol Biochem.

[CR45] Patriquin DG, Döbereiner J, Jain DX (1983). Sites and processes of association between diazotrophs and grasses. Can J Microbiol.

[CR46] Pereyra CM, Ramella NA, Pereyra MA, Barassi CA, Creus CM (2010). Changes in cucumber hypocotyl cell wall dynamics caused by *Azospirillum brasilense* inoculation. Plant Physiol Biochem.

[CR47] Piccinin GG, Braccini AL, Dan LGM, Scapim CA, Ricci TT, Bazo GL (2013). Efficiency of seed inoculation with *Azospirillum brasilense* on agronomic characteristics and yield of wheat. Ind Crop Prod.

[CR48] Piccinin GG, Dan LGM, Braccini AL, Mariano DC, Okumura RS, Bazo GL, Ricci TTE (2011). Agronomic efficiency of *Azospirillum brasileinse* in physiological parameters and yield components in wheat crop. J Agron.

[CR49] Puente ML, Garcia JE, Perticari A. Investigación aplicada de *Azospirillum* para su uso como promotor del crecimento em cultivos de interes agronômico. In: Cassán FD, Garcia de Salamone L, editor. *Azospirillum* sp.: cell physiology, plant interactions and agronomic research in Argentina. Argentina: Asociación Argentina de Microbiologia, Buenos Aires. 1994. pp. 167–178.

[CR50] Rodriguez H, Gonzalez T, Goire I, Bashan Y (2004). Gluconic acid production and phosphate solubilization by the plant growth-promoting bacterium *Azospirillum* spp. Naturwissenschaften.

[CR51] Romero AM, Correa OS, Moccia S, Rivas JG (2003). Effect of *Azospirillum* mediated plant growth promotion on the development of bacterial diseases on fresh-market and cherry tomato. J Appl Microbiol.

[CR52] Rothballer M, Schmid M, Hartmann A (2003). *In situ* localization and PGPB effect of *Azospirillum brasilense* strains colonizing roots of different wheat varieties. Symbiosis.

[CR53] Salantur A, Ozturk R, Akten S (2006). Growth and yield response of spring wheat (*Triticum aestivum* L.) to inoculation with rhizobacteria. Plant Soil Environ.

[CR54] Saubidet MI, Fatta N, Barneix AJ (2002). The effect of inoculation with *Azospirillum brasilense* on growth and nitrogen utilization by wheat plants. Plant Soil.

[CR55] SAS Institute (2001) Proprietary of software, version 8.2, 6th edn. SAS, Cary, USA.

[CR56] Searle PL (1984). The Berthelot or indophenol reaction and its use in the analytical chemistry of nitrogen. Analyst.

[CR57] Souza AO, Pamphile JA, Rocha CLMSC, Azevedo JL (2004). Plant-microbe interactions between maize (*Zea mays* L.) and endophytic microorganisms observed by scanning electron microscopy. Acta Sci Biol Sci.

[CR58] Sparks DL (1996). Methods of soil analysis, part 3, chemical method.

[CR59] Stancheva I, Dimitrov I, Kaloyanova N, Dimitrova A, Angelov M (1992). Effect of inoculation with *Azospirillum brasilense* on photosynthetic enzyme activities and grain yield in maize. Agronomie.

[CR60] Swedrzyńska D, Sawicka A (2001). Effect of Inoculation on population numbers of *Azospirillum* bacteria under winter wheat, oat and maize. Pol J Environ Stud.

[CR61] Tien TM, Gaskins MH, Hubbell DH (1979). Plant growth substances produced by *Azospirillum brasilense* and their effect on the growth of pearl millet (*Pennisetum americanum* L.). Appl Environ Microbiol.

[CR62] Tortora M, Diaz-Ricci JC, Pedraza R (2011). *Azospirillum brasilense* siderophores with antifungal activity against *Colletotrichum acutatum*. Arch Microbiol.

[CR63] Trani PE, Hiroce R, Bataglia OC (1983). Análise foliar: amostragem e interpretação.

[CR64] Venieraki A, Dimou M, Pergalis P, Kefalogianni I, Chatzipavlidis I, Katinakis P (2011). The genetic diversity of culturable nitrogen-fixing bacteria in the rhizosphere of wheat. Microb Ecol.

[CR65] Werner T, Motyka V, Laucou V, Smets R, Onckelen HV, Schmulling T (2003). Cytokinin-deficient transgenic *Arabidopsis* plants show multiple developmental alterations indicating opposite functions of cytokinins in the regulation of shoot and root meristem activity. Plant Cell.

[CR66] Yang C, Hamel C, Vujanovic V, Gan Y. Fungicides: modes of action and possible impact on nontarget microorganisms. ISRN Ecol. 2011; Article ID 130289 doi: 10.5402/2011/130289.

